# OphthoEvidence report: Comparative effects of prophylactic strategies for post-intravitreal injection endophthalmitis: Protocol for a systematic review and network meta-analysis

**DOI:** 10.1371/journal.pone.0354670

**Published:** 2026-07-28

**Authors:** Maryam Ghadimi, Dena Zeraatkar, João Pedro Lima, Jinhui Ma, Sunir Garg, Peter Kaiser, David Steel, Tien Yin Wong, Sobha Sivaprasad, Charles Wykoff, Varun Chaudhary

**Affiliations:** 1 Department of Health Research Methods, Evidence, and Impact, McMaster University, Hamilton, ON, Canada; 2 Division of Ophthalmology, Department of Surgery, McMaster University, Hamilton, ON, Canada; 3 Thomas Kevill Advanced Vitreoretinal Imaging (AVI) Lab, St Joseph’s Healthcare, Hamilton, ON, Canada; 4 Department of Anesthesia, McMaster University, Hamilton, ON, Canada; 5 The Retina Service of Wills Eye Hospital, Mid Atlantic Retina, Thomas Jefferson University, Philadelphia, Pennsylvania, United States of America; 6 Cole Eye Institute, Cleveland Clinic, Cleveland, Ohio, United States of America; 7 Bioscience Institute, Newcastle University, Newcastle Upon Tyne, United Kingdom; 8 Sunderland Eye Infirmary, Sunderland, United Kingdom; 9 Singapore Eye Research Institute, Singapore National Eye Centre, Singapore, Singapore; 10 Beijing Visual Science and Translational Eye Research Institute, School of Clinical Medicine, Beijing Tsinghua Changgung Hospital, Tsinghua Medicine, Tsinghua University, Beijing, China; 11 NIHR Biomedical Research Centre at Moorfields Eye Hospital and UCL Institute of Ophthalmology, London, United Kingdom; 12 Retina Consultants of Texas, Houston, Texas, Blanton Eye Institute, Houston Methodist Hospital, Houston, Texas, United States of America; Sanmenxia Central Hospital, Henan University of Science and Technilogy, CHINA

## Abstract

**Background:**

Endophthalmitis is a rare but serious complication of intravitreal injections (IVI). To mitigate the risk of post-injection endophthalmitis (PIE), retinal specialists use different strategies, combining various prophylactic measures. However, existing systematic reviews have evaluated the effects of these measures in isolation, which does not consider their concurrent use in practice. A trustworthy systematic review with network meta-analysis (NMA) by providing a framework for simultaneous comparisons of different prophylactic combinations is essential to guide decision-making.

**Objectives:**

To present a protocol for addressing the comparative effects of different strategies for the prevention of PIE.

**Methods:**

We will search Medline, EMBASE, Cochrane CENTRAL, Web of Science, and ClinicalTrials.gov from inception for randomized controlled trials and observational studies comparing any strategy for preventing PIE with an alternative strategy, or placebo, or no prophylactic strategy in patients aged 18 years or older who received IVI of any pharmacological agent for any indication except treatment of bacterial endophthalmitis. Paired reviewers will independently screen studies, extract the data and assess risk of bias for each outcome that includes infectious endophthalmitis, confirmed endophthalmitis, any endophthalmitis, need for surgical intervention for management of PIE, and best-corrected visual acuity. For each outcome, when possible, we will conduct frequentist random-effects NMAs. We will assess the certainty of evidence and interpret findings using the Grading of Recommendations Assessment, Development and Evaluation (GRADE) approach.

**Discussion:**

This systematic review and NMA will provide a comprehensive and up-to-date summary of the evidence addressing the effects of all available strategies for the prevention of PIE.

**Registration:**

Open Science Framework (https://doi.org/10.17605/OSF.IO/SJC4U).

## Introduction

Endophthalmitis is a rare but serious complication of intravitreal injections (IVI), increasingly important due to the expanding use of medications including anti-vascular endothelial growth factor (VEGF) agents, corticosteroids, anti-complement therapies, and other therapies in the current practice of ophthalmology worldwide [1]. The incidence of post-injection endophthalmitis (PIE) ranges from 0.02% to 0.09% per injection [2–4], with the cumulative risk per eye approaching 0.84% as patients usually receive multiple injections over their clinical course [5,6]. PIE presents as infectious or sterile intraocular inflammation [7], typically occurring 3–4 days after IVI [8]. PIE is associated with other possible complications, including secondary retinal detachment and irreversible vision loss [5,8].

There are several approaches to reduce the risk of PIE. However, the optimal prophylactic strategy remains uncertain. While ocular surface preparation with antiseptics (e.g., povidone–iodine, chlorhexidine) is a routine component of PIE prophylaxis [9], there is substantial variability in the use of other potential preventive measures. Across different surveys of retina specialists, 5% to 90% reported administering pre or post-injection antibiotic eye drops, 21% to 91% reported using a sterile eyelid speculum, 9% to 82% reported using sterile eye drape, 29% to 60% reported wearing masks during the procedure, 35% to 64% reported wearing sterile gloves, and 6% to 28% reported performing IVI in operating rooms [9–13].

Multiple systematic reviews have evaluated the effects of strategies for PIE prevention [14–19]. However, these studies focused on addressing the effects of individual prophylactic measures (e.g., comparing strategies with versus without topical antibiotics) that have been directly studied in randomized controlled trials (RCT) or observational studies. Given that various strategies, combining different sets of prophylactic measures, are being implemented in practice, network meta-analysis (NMA) provides a framework for simultaneous comparisons of these strategies, allowing estimation of the relative effects of strategies that lack direct comparative evidence [20]. We present a protocol for a systematic review and NMA to provide a comprehensive synthesis of evidence on comparative effects of all prophylactic strategies for PIE.

## Materials and methods

Our protocol adhered to the Preferred Reporting Items for Systematic Reviews and Meta-Analysis Protocols (PRISMA-P) guidelines (S1 Table) [21]. We registered this review in Open Science Framework [22] and will report our systematic review in accordance with the Preferred Reporting Items for Systematic Reviews and Meta-Analyses (PRISMA) with network meta-analysis extension [23,24]. Table 1 present the timeline of the review.

**Table 1:** Stages of the systematic review and timeline at protocol submission.

Stages of the review	Started	Anticipated completion date
Development of search strategy	Yes	October 20, 2025
Systematic search	No	October 31, 2025
Screening records	No	December 25, 2025
Data extraction	No	May 15, 2026
Risk of bias assessment	No	May 15, 2026
Data synthesis and analysis	No	June 30, 2026
Assessment of certainty of evidence	No	July 30, 2026
Interpretation of findings	No	August 15, 2026
Preparation of manuscript	No	August 20, 2026

### Eligibility criteria

We will include RCTs and observational studies comparing any strategy for preventing PIE with an alternative strategy or placebo, or no prophylactic strategy in patients aged 18 years or older who received IVI of any pharmacological agents in standard formulation or as sustained release implant for indications, including but not limited to diabetic macular edema, age-related macular degeneration, retinal venous occlusion, proliferative vitreoretinopathy, uveitis, intraocular lymphoma, and viral retinitis. We will exclude studies in which IVI was performed for the treatment of microbial endophthalmitis (e.g., IVI of antibiotics) or during a surgical procedure.

Eligible observational studies include those that prospectively enrolled or retrospectively identified participants according to their exposure to different prophylactic strategies (i.e., prospective or retrospective cohort) as well as studies that sampled participants based on the occurrence of PIE from a defined cohort of all patients who received IVI over a certain period (i.e., nested case-control). To limit the inclusion of observational studies that are too small to provide reliable estimates, considering the incidence of PIE, we will exclude observational studies with fewer than 1000 injections across all arms.

A prophylactic strategy can be performed before, during, or immediately after IVI and includes one or combinations of any of the following components: preparing ocular surface with topical antiseptics, administering antibiotic eye drops, using a sterile eye drape, using a sterile eyelid speculum, wearing sterile gloves, universal (physician and patient) face masking, physician-only face masking, adopting a no-talking policy, and performing the procedure in an operating room. Eligible studies compare at least two strategies that differ in at least one of these components. This also includes studies that compared different antiseptic agents or antibiotics. We will include studies regardless of language, year of publication, and publication status.

### Search strategy and study selection

With the help of an experienced medical librarian, we will develop specific search strategies and will search Medline, EMBASE, Cochrane CENTRAL, Web of Science, and ClinicalTrials.gov from inception for eligible studies. We will review the reference lists of the included studies and relevant systematic reviews for potentially eligible studies not captured by our search. Using Covidence [25], pairs of reviewers will independently screen titles and abstracts of retrieved citations, followed by full texts of potentially eligible studies. Reviewers will resolve discrepancies by discussion or by adjudication with a third reviewer.

### Data extraction

Following calibration exercises and using standardized, pilot-tested data extraction forms, pairs of reviewers will independently extract data from included studies. Reviewers will resolve discrepancies by discussion or by adjudication with a third reviewer. From each eligible study, we will extract information on study characteristics (e.g., year, country of origin, sources of funding), patient characteristics (e.g., age, sex, indication for IVI, mean number of cumulative IVI received, mean days to PIE presentation), details of IVI procedure (e.g., unilateral or same-day bilateral injection, type of drug injected, prefilled syringe or conventional preparation, type of anesthesia), details of prophylactic strategies and comparators (e.g., type of antiseptics and antibiotics, timing of exposure to antiseptics, timing of antibiotic prophylaxis, using nose tape for patient masks), and outcomes of interest. For studies that do not explicitly report on any component of the prophylactic strategy, we will contact the corresponding authors to obtain the missing information. If no response is received, we will assume that the unreported components were not implemented in that study arm.

We will classify infectious endophthalmitis, defined as any endophthalmitis event that occurred post-IVI and necessitated treatment with intravitreal antibiotics, as a critical outcome. Important outcomes include microbiologically confirmed endophthalmitis (defined as culture or Gram stain positive endophthalmitis events), any endophthalmitis (i.e., all clinically suspected endophthalmitis events), need for surgical intervention for management of PIE, and best-corrected visual acuity (BCVA) among patients who developed PIE. When authors reported outcomes at multiple timepoints, we will use the longest available follow-up. For dichotomous outcomes reported in RCTs, reviewers, for each arm, will extract number of injections and events for endophthalmitis and number of patients and events for need for surgical intervention or, for observational studies, if available, adjusted relative effects and corresponding 95% confidence intervals (CI) per injection for endophthalmitis, and per patient for need for surgical intervention. For studies that only report endophthalmitis events per patient, if available, we will extract the mean number of injections per patient to calculate the number of injections for each arm. For BCVA, we will extract mean or median visual acuity and measure of variability or, for observational studies, if available, adjusted mean difference (MD) and corresponding 95% CI.

For observational studies, we will prioritize relative effects accounted for correlation in outcomes from injections in the same eye and the same patient. As suggested by studies that evaluated risk factors for PIE, we will consider the following confounders for outcomes of interest: sex, diabetes, presence of blepharitis, cumulative number of prior injections, type of drug injected, prefilled syringe, type of anesthesia, patient squeezing during the injection, and use of a disposable conjunctival mould assist device [5,8,26–29]. To minimize the risk of bias due to confounding [30], if observational studies report multiple adjusted effect estimates for the same comparison, we will extract the estimate from the model with the most comprehensive adjustment for pre-specified important confounding domains (i.e., cumulative number of prior injections, type of drug injected, prefilled syringe, type of anesthesia). When multiple models are comparable in this regard, we will select the model that additionally adjusts for the greatest number of additional confounding domains (i.e., sex, diabetes, presence of blepharitis, patient squeezing during the injection, and use of a disposable conjunctival mould assist device). For randomized controlled trials, we will extract outcome data from the intention-to-treat (ITT) population and, if not reported, in preferred order, modified-ITT, per-protocol, and as-treated population.

### Risk of bias assessment

For each eligible study and each outcome, paired reviewers, following calibration exercises, will independently assess risk of bias and will resolve discrepancies by discussion with a third reviewer. For RCTs, we will use the Risk Of Bias instrument for Use in SysTematic reviews – for Randomised Controlled Trials (ROBUST-RCT) [31]. In two steps, reviewers will first assess the implementation of methodological safeguards against biases related to the sequence generation process, allocation concealment, blinding of patients, blinding of healthcare providers, blinding of outcome assessors, and missing outcome data. Then, reviewers will judge the risk of bias based on any deficits in the safeguards. Reviewers will rate risk of bias as “definitely low risk of bias”, “probably low risk of bias”, “probably high risk of bias”, and “definitely high risk of bias”.

For observational studies, we will use Risk of Bias In Non-randomised Studies – of Interventions (ROBINS-I) instrument [32], addressing biases due to confounding, in selection of participants into the study, in classification of interventions, due to deviation from the intended interventions, from missing outcome data, in measurement of the outcome, and in selection of the reported results. The ROBINS-I tool classifies risk of bias as either “low”, “moderate”, “serious”, or “critical”. When all the domains for ROBUST-RCT are definitely low or probably low risk and for ROBINS-I are low risk, we will rate the overall risk of bias of the study as “low”; otherwise, we will rate the overall risk of bias as “high”.

### Data synthesis and analysis

To describe data on characteristics of studies, participants, IVI procedure, and prophylactic strategies, we will use median and interquartile range for continuous variables and frequency and proportions for categorical variables. We will pool dichotomous outcomes using the odds ratio (OR) and associated 95% CI. For BCVA, we will calculate MD and corresponding 95% CI. We will convert BCVA values reported in studies based on Snellen fraction or the logarithm of the minimum angle of resolution to the more intuitive alternative score, the approximate Early Treatment Diabetic Retinopathy Study letter, according to Gregori et al [33].

We will classify treatment nodes based on the class of components of prophylactic strategies (i.e., antiseptic, antibiotic, sterile eyelid speculum, sterile eye drape, sterile gloves, universal masking, physician-only masking, no-talking policy, operating room). Each treatment node will represent a single component or a combination of different components. We will conduct secondary analyses separating nodes based on antiseptic type (i.e., povidone iodine, chlorhexidine, hypochlorous acid), antibiotic class (i.e., fluoroquinolones, other classes), and timing of antibiotic prophylaxis (i.e., pre-IVI, post-IVI, pre- and post-IVI). If we find evidence that the effects are different based on these factors, we will separate corresponding nodes for the final model.

We will conduct NMA for each outcome, separately for RCTs and observational studies [30], informed by at least 10 studies. If for at least 20% of comparisons of an outcome, evidence is downgraded two times due to imprecision, we will combine RCTs and observational studies and base our conclusions on evidence from RCTs, observational studies, or their combination that provides the highest certainty. If NMA is not feasible (i.e., disconnected networks or few available studies), we will perform pairwise meta-analysis for comparisons informed by at least two trials [34]. If quantitative synthesis is not possible, we will descriptively present results from each included study. To facilitate interpretation of the results for dichotomous outcomes, we will calculate risk difference (RD) per 1000 injections using the baseline risk derived from the median risk of an event across the reference prophylactic strategy arms, which, for each network, is the strategy most connected to others.

For dichotomous outcomes from RCTs, we will conduct frequentist random effects NMA, assuming a common heterogeneity across all comparisons, under the generalized linear mixed models (GLMM) using a multivariate non-central hypergeometric likelihood [35]. To obtain direct estimates, we will perform random effects pairwise meta-analyses using the Hypergeometric-Normal models [35]. For dichotomous outcomes from observational studies and BCVA, we will conduct frequentist random-effects NMA, assuming a common heterogeneity across all comparisons under the multivariate meta-analysis models using the restricted maximum likelihood estimator [36] and will synthesize direct estimates using the DerSimonian–Laird random-effects pairwise meta-analyses [37]. When observational studies report relative risk (RR) rather than OR, we will use RR as an approximate estimate of OR, given that these measures are nearly similar for rare events [34]. For observational studies without estimates adjusted for confounders, we will apply the same approach as described for RCTs to pool raw dichotomous outcome data. We will exclude nested case-control studies that do not report adjusted effect estimates from the analysis and will descriptively present their results. As secondary analyses to explore the effects of individual components of the prophylactic strategy, assuming that the effect of the prophylactic strategy is the sum of the effects of its components, we will perform frequentist random effects component NMAs [38].

We will use design-by-treatment models and Wald’s test to assess incoherence at the entire network level [39] and the node-splitting approach to assess incoherence within each closed loop of evidence and to obtain indirect estimates [40]. If important incoherence exists in the network, we will explore potential sources of incoherence, including considering our a priori subgroup hypotheses for effect modification: (i) we hypothesized that studies with patients who received IVI for inflammatory versus other indications would report smaller effects; (ii) we hypothesized that studies with patients received IVI with immunosuppressive effects such as corticosteroids versus anti-VEGF agents or other drugs would report smaller effects; (iii) we hypothesized that studies used conventional preparation versus prefilled syringe for IVI would report smaller effects; (iv) we hypothesized that studies at low versus high risk of bias would report smaller effects. Network meta-regression will test effect modification by these variables. We will use similar a priori hypotheses to explore potential inconsistency in results between studies informing each direct comparison [41,42]. We will consider the similarity of point estimates, overlap of confidence intervals, I^2^ statistics, and the target of the certainty rating for judgment on inconsistency [41]. If we identify effect modification with moderate to high credibility that explains incoherence or inconsistency, we will conduct separate analyses stratified by subgroups; otherwise, we will rate down the certainty of evidence [41,43]. We will assess the credibility of any apparent effect modification (p-value for test of interaction < 0.05) using the Instrument to assess the Credibility of Effect Modification Analyses (ICEMAN) [44].

To assess publication bias, we will use the contour-enhanced funnel plot for each comparison informed by 10 or more studies to visually assess asymmetry in the funnel plots [45] and will test asymmetries in the funnel plots according to Eager et al [46].

We will perform a sensitivity analysis excluding studies that used topical anesthesia gel prior to the application of antiseptics on the ocular surface. To assess the robustness of our findings, we will conduct sensitivity analyses using fixed effects GLMM and fixed and random effects penalized likelihood [47] as an alternative to random effects NMA under GLMM. Because some observational studies may report the effects of multiple overlapping prophylactic strategies drawn from the same dataset, we will conduct sensitivity analyses retaining only the study with the largest number of events among studies with overlapping arms to avoid double-counting of individuals across studies and examine the robustness of the results. We will perform all analyses using the meta and netmeta packages in R (V.4.1.2, Vienna, Austria).

### Assessment of the certainty of the evidence

We will assess the certainty of the evidence using the Grading of Recommendations Assessment, Development, and Evaluation (GRADE) approach [48,49] with the target of certainty of a non-zero effect [50] (i.e., strategy A has a non-zero effect compared to strategy B) and will rate the certainty for each comparison and outcome as “high”, “moderate”, “low”, or “very low”. Two methodologists will rate each domain for each comparison per outcome, discussing uncertainties with a third methodologist.

The certainty of evidence for each direct estimate starts as high for RCTs and low for observational studies, and we will rate down for risk of bias [51], inconsistency [41,42], indirectness [52], and publication bias [53]. We will consider rating up the certainty of evidence for observational studies for a large magnitude of effect or when plausible residual confounding would further support inferences regarding the estimated effects [54]. For indirect estimates, the certainty starts as the lowest certainty of the contributing direct evidence to the most dominant first-order loop, with further rating down for intransitivity [55] if present. To assess intransitivity, we will examine whether important differences in the distribution of credible effect modifiers between the direct comparisons informing an indirect estimate exist. We will use similar a priori hypotheses for effect modifications as specified for exploring sources of incoherence and inconsistency. The certainty of network estimates is based on the direct or indirect evidence that most contributed to the network estimate, with further consideration of rating down for incoherence between direct and indirect estimates [43] and imprecision of network estimates [56]. We will rate down for imprecision if the CI crosses the null threshold. However, if the point estimate is near the null, suggesting that there is no difference between strategies, we will rate certainty in little or no difference and will rate down for imprecision if the CI crosses the minimal important difference (MID) threshold rather than the null. Clinical experts in the team will reach consensus on MID thresholds for each outcome.

### Interpretation of findings

Our interpretation of findings will be based on the GRADE approach [57] using MID thresholds to decide whether the effect of one strategy differs from another. First, if the CI comparing a strategy to the reference strategy crosses MID, we will classify the strategy in the same group as the reference. If the CI comparing a strategy to the reference does not cross MID, we will classify the strategy as more or less effective than the reference, depending on the direction of the effect. Subsequently, we will compare the strategies classified as more effective than the reference against each other by examining whether the CI of their estimated effects crosses the MID. Finally, we will separate strategies according to the certainty of the evidence. We will limit the classification to strategies with high, moderate, or low certainty evidence compared to the reference.

### Dissemination of findings

We will disseminate our findings by publication in a peer-reviewed journal and by presentation at national or international conferences. Drawing on the results of our review, we will develop network graphs for each outcome depicting direct and indirect comparisons between different prophylactic strategies, a colour-coded table classifying different prophylactic strategies across outcomes, and league tables for each outcome reporting relative and absolute direct, indirect, and network estimates per comparison.

## Discussion

Our systematic review and NMA will provide a clinically useful and trustworthy summary of the evidence addressing prophylactic strategies for PIE. This study is the first systematic review exploring the comparative effects of all available strategies. Other systematic reviews focused on pairwise comparisons and evaluated prophylactic strategies with versus without topical antibiotics [16–18], with povidone iodine versus chlorhexidine (as antiseptics) [14], and procedures performed in an operating room versus office-based setting [15].A recent systematic review and NMA only investigated different face masking policies for preventing PIE [19]. These reviews suggested no meaningful difference in the risk of PIE when comparing povidone-iodine versus chlorhexidine or operating room versus office-based settings, a higher risk of PIE associated with antibiotic prophylaxis, and a lower risk associated with physician masking and no-talking policy.

The strengths of this review include a comprehensive and up-to-date search strategy; inclusion of both RCTs and comparative observational studies; addressing patient-important outcomes; screening of studies, extracting data, and assessing risk of bias in duplicate to minimize bias; and application of GRADE methodology to assess the certainty of evidence and to classify prophylactic strategies from the best to the worst.

This review also has limitations. First, we anticipate limited direct evidence comparing different strategies with a low number of events, particularly from RCTs, which in turn may result in low or very low certainty of evidence for the effects of most strategies. Second, numerous factors may influence the choice of prophylactic strategies and the risk of occurrence of PIE (i.e., confounders). We anticipate that few observational studies would appropriately account for these factors; this would introduce a risk of bias issue and may also result in inconsistency between the results of observational studies. We will pool relative effects adjusted for prespecified confounders to minimize bias. Third, we will explore effect modification by potential effect modifiers. Because studies may report few events, it is possible that subgroup analyses would have limited statistical power to detect any meaningful findings. Fourth, as primary analysis for observational studies which did not report adjusted effect estimates and for RCTs, we will use the GLMM NMA approach. While this approach proved appropriate in simulation studies for NMA of rare events [35], this method excludes studies with zero events on all arms from the analysis and may lead to disconnected networks and influence the effect estimates. Therefore, to explore the robustness of the results, we will conduct sensitivity analyses using the penalized likelihood NMA method, which incorporates information from zero-event studies [47].

Our findings will inform retina specialists regarding the most effective strategies for the prevention of PIE and will provide a rigorous systematic review of evidence for professional societies that intend to develop guidelines focusing on patient care in IVI practice.

## Supporting information

S1 Table PRISMA-P (Preferred Reporting Items for Systematic review and Meta-Analysis Protocols) 2015 checklist. (DOCX)
